# A Soft Pressure Sensor Array Based on a Conducting Nanomembrane

**DOI:** 10.3390/mi12080933

**Published:** 2021-08-06

**Authors:** Daekwang Jung, Kyumin Kang, Hyunjin Jung, Duhwan Seong, Soojung An, Jiyong Yoon, Wooseok Kim, Mikyung Shin, Hyoung Won Baac, Sangmin Won, Changhwan Shin, Donghee Son

**Affiliations:** 1 Department of Electrical and Computer Engineering, Sungkyunkwan University, Suwon 16419, Korea; biglight0308@gmail.com (D.J.); zseqqq@gmail.com (K.K.); dodoworld1993@gmail.com (D.S.); soojung2134@gmail.com (S.A.); jiyong428@g.skku.edu (J.Y.); wooseokkim@skku.edu (W.K.); hwbaac@skku.edu (H.W.B.); sangminwon@skku.edu (S.W.); 2 School of Mechanical Engineering, Sungkyunkwan University, Suwon 16419, Korea; hyunjin.jung33@gmail.com; 3 Department of Intelligent Precision Healthcare Convergence, Sungkyunkwan University, Suwon 16419, Korea; mikyungshin@g.skku.edu; 4 Center for Neuroscience Imaging Research, Institute for Basic Science (IBS), Suwon 16419, Korea; 5 Department of Superintelligence Engineering, Sungkyunkwan University, Suwon 16419, Korea

**Keywords:** pressure sensor, stretchable electronics, piezoresistive, electronic skin

## Abstract

Although skin-like pressure sensors exhibit high sensitivity with a high performance over a wide area, they have limitations owing to the critical issue of being linear only in a narrow strain range. Various strategies have been proposed to improve the performance of soft pressure sensors, but such a nonlinearity issue still exists and the sensors are only effective within a very narrow strain range. Herein, we fabricated a highly sensitive multi-channel pressure sensor array by using a simple thermal evaporation process of conducting nanomembranes onto a stretchable substrate. A rigid-island structure capable of dissipating accumulated strain energy induced by external mechanical stimuli was adopted for the sensor. The performance of the sensor was precisely controlled by optimizing the thickness of the stretchable substrate and the number of serpentines of an Au membrane. The fabricated sensor exhibited a sensitivity of 0.675 kPa^−1^ in the broad pressure range of 2.3–50 kPa with linearity (~0.990), and good stability (>300 Cycles). Finally, we successfully demonstrated a mapping of pressure distribution.

## 1. Introduction

E-skin [1,2,3,4,5,6,7,8,9,10], human–machine interfacing (HMI) [11,12,13,14], wearable healthcare devices [15,16,17,18], and soft robotics [19,20,21] that can transmit external stimuli applied to the skin as electrical signals, are receiving widespread attention in various fields. These devices require sensors that can transmit external stimuli as electrical signals. However, it is difficult for these sensors to obtain stable signals because of their difference in modulus with the skin. Therefore, the focus is on realizing a flexible and stretchable device that can overcome this difference in modulus.

Among such flexible and stretchable sensors, pressure sensors have enormous potential and adaptability to various devices. They are used in healthcare devices because they can provide vital bio-signals such as blood pressure and skin deformation with high reliability and non-invasiveness. These sensors consist of four types: 2D material type [5,22,23], capacitance type [7,24,25,26], piezoelectric type [27,28], or piezoresistive type [29,30,31,32]. Among these types, the advantages of the capacitance-type pressure sensor include a simple pressure derivation formula, low power consumption, and relatively high sensitivity. However, because the area occupied by the sensor affects the signal-to-noise ratio (SNR), and the noise interference from various external stimuli is severe, it is difficult to achieve multi-channel mode and miniaturization [33,34]. The piezoelectric-type pressure sensor converts normal force into electrical charge using inorganic piezoelectric materials such as lead zirconate titanate (PZT) and ZnO. Its advantages include a high sensitivity to dynamic pressure and fast response. Conversely, errors may be induced by the presence of signal drift, and its measurement properties have a low reliability [35,36,37]. An additional feedback loop system is needed to solve this problem, which leads to a poor measurement modality. The piezoresistive pressure sensor has a simple structure, so its manufacturing process is also simple. Additionally, its signal readout mechanism and formula are simple, so easy microfabrication is possible. In addition, the stretchable piezoresistive pressure sensor can measure both dynamic and static pressure, and because of its high sensitivity and high linearity over a wide pressure range, it is desirable for acquiring the physiological pressure data of the human body. Therefore, in order to overcome the difference in modulus with the skin, various strategies with piezoresistive pressure sensors were proposed. Among them, a pressure sensor with high sensitivity was proposed by making microstructures such as micropyramids [10,31,38,39], microdomes [40], and hollow spheres [41] on stretchable dielectric layers. These strategies all essentially have high sensitivity. However, the fabrication process is relatively difficult, and it has low linearity due to the gradual deformation in the high-pressure region [31]. As another strategy, a pressure sensor made to be stretchable using a porous substrate like a sponge has been proposed [32,42]. However, these strategies have a limited strain range, and the pre-existing space affects the sensitivity in the low-pressure region [32]. It is important to have high linearity both in the high-pressure region and in the low-pressure region.

**Figure 1:**
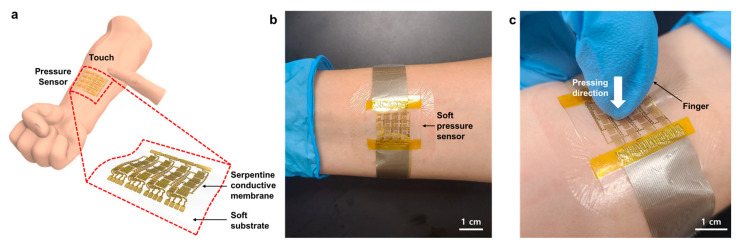
(**a**) Overall schematic diagram of the soft pressure sensor array. (**b**) Photograph of the device placed on the skin. (**c**) Photograph of the device pressed after placing on the skin.

Herein, we propose a soft pressure sensor array based on a conducting nanomembrane for human–machine interfaces. Our device shows high linearity over a wide pressure range using a serpentine rigid-islands structure with a Au nanomembrane. Since microstructure is not required, the microfabrication process is quite simple and has relatively high sensitivity. The stretchability of the device was ensured by using a highly stretchable styrene–ethylene–butylene–styrene (SEBS) substrate. The performance of the devices was precisely controlled by optimizing the thickness of the stretchable substrate and the number of serpentines in the Au nanomembrane. The fabricated devices, by using a simple thermal evaporation process, exhibited a sensitivity of 0.675 kPa^−1^ in the broad pressure range of 2.3–50 kPa with linearity (~0.990) and good stability (>300 Cycles). Furthermore, a pressure sensor array has been demonstrated for the mapping of pressure distribution. As shown in Figure 1a–c, our device can be used as a multi–channel pressure sensor array in e–skin due to its soft property.

## 2. Materials and Methods

### 2.1. Simulation of Stretchable Pressure Sensor

Since the value of the pressure sensor depended on the strain of the Au nanomembrane, it was important to understand the pressure-induced deformation of sensors. When the Au nanomembrane was pressed, the electrical resistance changed. Under the specific pressure, the substrate thickness and deformation affected the sensitivity of the sensor. Essentially, thinner substrates were more deformed and more sensitive. However, a substrate that is too thin may cause a larger deformation of the Au nanomembrane due to its poor strain dissipation ability. As a result, the sensor with a very thin substrate has limited operating pressure ranges because of irreversible damage to the Au nanomembrane [43]. With the effect of the substrate thickness, the proportion of the actual pressed area to the entire serpentine area of the sensor affects the strain value of the Au nanomembrane. This is because the wider the pressed area, the greater the deflection of the contact edge of the gold serpentine nanomembrane by the rigid and flat cylindrical bar. Thus, it is important to systemically study the sensing mechanism, including the effect of the substrate thickness on the sensor performance and the pressure-induced strain on the serpentine area using simulation.

**Figure 2:**
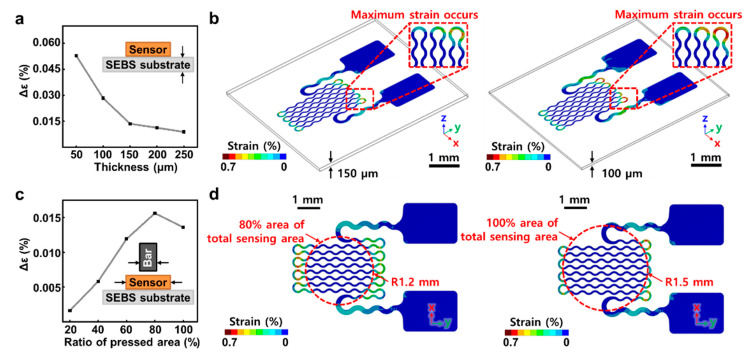
Finite element Analysis (FEA) simulation showing the pressure-induced strain in sensors with different SEBS substrate thickness and pressured area to the total area (**a**) Maximum von Mises strain shift plotted according to the pressure in respect to the pressed area ratio to total sensing area when the SEBS substrate is 150 μm thick. (**b**) Contour profile of the maximum von Mises strain in two types of sensors (80% and 100% ratio of the sensing area) when a pressure of 25 kPa was applied perpendicular to the sensor surface. (**c**) Maximum von Mises strain shift plotted according to the pressure with respect to the SEBS substrate thickness. (**d**) Contour profile of the maximum von Mises strain in two types of sensors (substrate thickness of 150 and 100 μm) when a pressure of 25 kPa was applied perpendicular to the sensor surface.

To understand the deformation of the sensor by external pressure and the strain of the Au nanomembrane, we used engineering simulation software (ANSYS, Ansys Inc., Canonsburg, PA, USA) to simulate the finite element analysis (FEA) on the effects of the reference temperature (stress-free) as shown in Figure 2. The simulation calculated the deformation of the Au nanomembrane encapsulated with PI (Poly (pyromellitic dihydride-co-4,4′-oxydianiline) and SEBS materials and, based on the results, the von Mises-Hencky theory (also known as the shear-energy theory or maximum distortion energy theory) was utilized. To compare the strain changes with the substrate thickness, five different types of SEBS substrate (thickness of 50, 100, 150, 200, and 250 μm) were designed to conduct contact assembly with the model. Figure 2a shows that samples of thin substrates have a larger maximum von Mises strain change, compared to the samples of thicker substrates, when pressed with the cylindrical bar on a full dimension of the serpentine area. However, a rapid increase in strain changes below 100 μm thickness caused irreversible damage to the Au nanomembrane. Figure 2b is a contour image comparing the von Mises strain in the Au nanomembrane on 150 μm and 100 μm substrates. Unlike the strain on the 150 μm substrate, a critical strain occurred at the edge where the Au nanomembrane and the cylindrical bar were contacting, and we analyzed that the poor strain dissipation ability was shown on the 100 μm and the thinner substrate. To compare how the pressure with different areas affected the amount of strain changes, the substrate thickness was fixed to 150 μm and five different diameters (20%, 40%, 60%, 80%, and 100% ratio of bar with 3 mm bottom) were designed with a flat and high-strength ABS (Acrylonitrile butadiene styrene) cylindrical bar. Subsequently, the simulation was performed by contact assembly with the pressure sensor model of the serpentine structure. Figure 2c shows that the wider the ratio of pressed serpentine area to the total area, the greater the amount of maximum von Mises strain change in the Au nanomembrane. Specifically, the strain became critical when pressed with a cylindrical bar with a diameter of more than 80% of the total area. When pressure was applied to 80% of the total area of the sensing area, a larger strain occurred at the contact edge, but irreversibly damaged the gold membrane [44,45]. Therefore, it is estimated that a critical strain will occur when pressure is applied to the entire area and that it will exhibit a relatively stable repeatability than the former case, as shown in Figure 2d. From the FEA result, we set the condition to give the sensor above 150 μm substrates as much pressure as the entire area of our pressure sensor design rule and predicted that it would have the best sensitivity. Section 2.2 explains how to fabricate the sensor designed with the above FEA results.

### 2.2. Fabrication and Transfer Printing of a Stretchable Pressure Sensor

**Figure 3:**
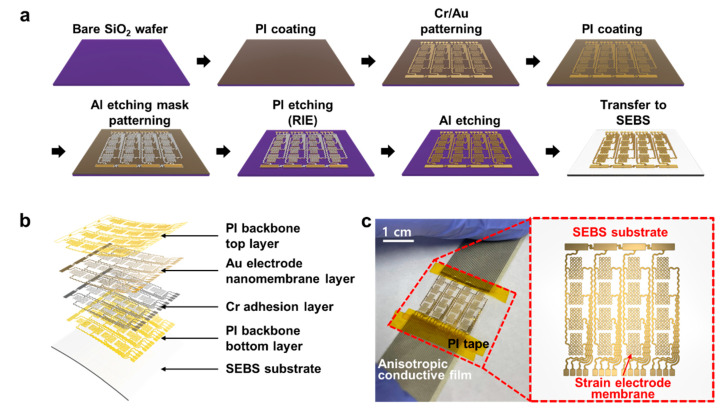
Schematic illustrations and photographic image of the soft pressure sensor (**a**) Fabrication process of the soft nanomembrane pressure sensor. (**b**) The pressure sensor consisting of a SEBS substrate (bottom white)—PI bottom layer (bright yellow)—Cr adhesive layer (grey)—Au nanomembrane layer (dark yellow)—PI top layer (bright yellow). (**c**) A photograph of a stretchable pressure sensor.

Figure 3a shows the fabrication process of the soft pressure sensor electrode. A 4 × 4 array of pressure sensor electrodes consists of a single electrode component with a 7.5 mm × 7.5 mm^2^ structure. The interconnection of each electrode consists of a serpentine structure, enabling stretchability in the overall system deformation via mechanical strain. The fabrication process of the metal-based electrodes was carried out on a SiO_2_ wafer substrate, and the electrodes had a bilayer structure consisting of a chromium (Cr) adhesion layer and a gold (Au) membrane layer. To separate the electrode from the wafer substrate, the top and bottom sides of the electrodes were supported with polyimide (PI) backbone layers. PI, amic acid solution 12.8 wt%, Sigma-Aldrich, Burlington, MA, USA) solution was uniformly coated with a spin-coater (SPIN-3000D, MIDAS SYSTEM Co., Ltd., Daejeon, Korea) on a treated substrate and baked in an oven. When the bottom layer of the PI backbone was formed on the wafer, Cr/Au nanomembrane layers were deposited using a thermal evaporator (A-SYSTEMS). Subsequently, a positive PR (GXR-601, AZ Electronic Materials, Luxembourg) was spin coated, and the photolithography process was performed using a mask aligner (MDA-400M, MIDAS SYSTEM Co., Ltd.) to draw electrode patterns on the substrate. A developer (MIF-300, AZ Electronic Materials, Luxembourg) was used to remove the exposed PR, resulting in electrode patterns. Afterward, the electrode patterns were left on the substrate using Au etchant (TFA, Transene Company, Inc., Danvers, MA, USA) and Cr etchant (CE-905N, Transene Company, Inc., Danvers, MA, USA) in order, and the wafer was immersed in acetone (Sigma-Aldrich, Burlington, MA, USA) to remove the undeveloped PR. A PI layer was coated once again and baked to encapsulate the electrode pattern. An aluminum (Al) layer was used as an etching mask to draw patterns on the PI backbone layers and open the sensing area of the electrode layer. On a PI-coated wafer, Al was deposited using a thermal evaporator, the same PR used previously was coated, and a photolithography process was performed to draw an etching mask pattern. The developer then formed an Al etching mask pattern; subsequently, the wafer was immersed into an Al etchant (APAL-1, LABOTECH Co., Ltd., Daejeon, Korea), leaving the pattern intact, and then immersed in acetone, leaving only the Al layer on the PI backbone. The reactive ion etching (RIE; PlasmaLab system 80 RIE, Oxford Instruments, Abingdon, UK) was performed to remove the exposed PI area. During the process, the Al layer on the PI backbone and the Cr/Au membrane layers functioned as an etching mask to protect the PI layer below. Subsequently, the wafer was immersed into the Al etchant to remove the remaining Al layer, and the microfabrication process of the electrode devices was completed. Further details of the fabrication process are described in Supplementary Note 1.

After the fabrication of the electrode device, the transfer-printing method was applied to move the device from the wafer to the stretchable SEBS (Tuftec^TM^ H1062, Asahi Kasei Co., Tokyo, Japan) substrate. A piece of Teflon tape (903UL, Nitto Co., Ltd., Tokyo, Japan) was attached to the slide glass so that the SEBS substrate could be well separated from the glass, and the SEBS solution (75 mg/mL in chloroform) was uniformly drop-casted and cured overnight. Using water-soluble tape (No. 5414, 3M Co., Ltd., Maplewood, MN, USA), the stretchable pressure sensor was removed from the wafer and transferred to the SEBS substrate. The water-soluble tape was dissolved in deionized water, and the transfer printing of the stretchable pressure sensor electrode to the elastomer substrate was completed. Figure 3b shows each element layer of the finished stretchable pressure sensor electrode patch. There is a SEBS stretchable substrate below, above which the Au membrane was encapsulated as a PI backbone with a Cr adhesion layer. Figure 3c shows a photographic image of the electrode sample.

## 3. Results

### 3.1. Single-Cell Pressure Sensor Test

**Figure 4:**
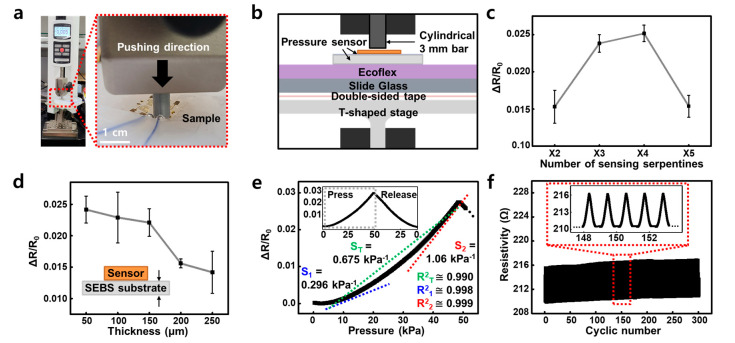
Experimental results of the prototype pressure sensor (**a**) A photograph of the experimentation. (**b**) Schematic diagram of the experimental method (Scale bar: 1 cm). (**c**) Relative resistance change of the pressure sensor according to the number of serpentine electrodes and (**d**) thickness of substrate. (**e**) Relative resistance changes of the pressure sensor according to pressure and 1 cycle of pressing and releasing (inset). (**f**) Cyclic test of the targeted pressure sensor and close-up view of cyclic pressure sensing at middle range (inset).

A press test was performed to measure the change in the resistance value of the soft pressure sensor fabricated in Section 2. All measurements were taken in an environment with a humidity of 68% and a temperature of 23 °C. Figure 4 shows the press test of the prototype pressure sensor fabricated by the process in Figure 3. Press test (Figure 4a) was performed by using the motorized force tester (ESM303, Mark-10 Co., New York, NY, USA) to press the sample with a cylindrical bar. Figure 4b is a schematic diagram showing the experimental setup as a side view. The slide glass substrate was fixed on the chuck clamp of the motorized force tester by the T-shaped stage and double-sided tape, and EcoFlex (Ecoflex^TM^ 00–10, Smooth-on Inc., Macungie, PA, USA) with a thickness of 1 cm to mimic the skin modulus. The press test was carried out with the pressure sensor on the top. Each sample was transferred to a 1.5 cm × 1.5 cm dimension SEBS substrate, and a source meter (2450 Digital Multimeter, Keithely, Solon, OH, USA) was used to measure relative resistance changes. Additionally, the pad of the device and the annealed copper wire were contacted with liquid metal (EGaIn, Sigma-Aldrich, Burlington, VT, USA). The press test was performed with a 3 mm cylindrical bar at a speed of 12 mm/min.

To optimize the highly sensing property of the serpentine structure-based pressure sensor, various design rules were used. First, to optimize the number of sensing serpentines, pressure sensors of various lengths were prepared (×2, ×3, ×4 and ×5 patterns). The SEBS substrate of each sample was fixed to a thickness of 150 μm, a cylindrical bar diameter of 3 mm, a pressing and releasing speed of 12 mm/min, and a deform range by pressing to 2.75 mm. Because the 3 mm diameter bar was designed to correspond to 80% of the width of the ×4 sensing serpentine, the ×1~×3 length sample had the effect of pressing with more than 80% of the object dimension, and the ×5 length sample had an object dimension of 80% or less at same 3 mm diameter bar. As a result of the experiment, the change of relative resistance was the highest at approximately 0.025 in the ×4 pattern, and lowest at approximately 0.0155 in the ×2 and ×5 patterns (Figure 4c and Figure S1), the same result as predicted in Figure 2a was confirmed by a real device. Based on this, ×4 sensing serpentines of the pressure sensor were selected. In addition, to optimize the thickness of the substrate, SEBS substrates of various thicknesses were manufactured (50, 100, 150, 200 and 250 μm). Each of the samples had ×4 sensing serpentines, and were tested under the same conditions as the sensing serpentine optimization. As a result of the experiment, the relative resistance change was validated to be the highest at approximately 0.024 at 50 μm thickness, 0.015 at 200 μm thickness, and 0.014 at the lowest thickness of 250 μm (Figure 4d and Figure S2). As mentioned in Figure 2, the thinner the substrate, the higher deformation and sensitivity can be secured. However, if the substrate is too thin, the strain dissipation ability decreases when pressure is applied, causing greater deformation of the sensing serpentine. In the end, damage to the Au nanomembrane structure made it impossible to repeatedly operate. Therefore, from the result of Figure 4d, compared with ΔR/R_0_ with a thickness of 50 μm, normalized resistance was retained and the thickness was set to 150 μm, the thinnest possible. As a result, it was confirmed that the prototype sensor had the same tendency as predicted by the FEA simulation, and based on this, the serpentine dimensions and substrate thickness of the most efficient pressure sensor were selected.

Sensitivity of the prototype pressure sensor manufactured through these design rules was calculated through the results of one cycle (Figure 4e) and 300 cyclic operations (Figure 4f). However, fatigue of the SEBS substrate occurs due to hundreds and close to 1000 cyclic tests, and the results in Figure 4f and Figure S3 are confirmed. As in the previous experiments (Figure 4c,d), the pressing and releasing speed was 12 mm/min, the cylinder diameter was 3 mm, and the deform change was 2.75 mm. The sensitivity of the single cell was confirmed to have a maximum sensitivity of 1.06 kPa^−1^ (S_2_) and 0.296 kPa^−1^ (S_1_); near 10 kPa when applied pressure was constantly increased up to 50 kPa. In addition, the device had a sensitivity of 0.675 kPa^−1^ (S_T_) from 10 kPa to 50 kPa. The 10 kPa, 10 to 50 kPa and 50 kPa linearity were 0.998 (R^2^_1_), 0.999 (R^2^_2_), and 0.990 (R^2^_T_), respectively. It was also confirmed that the linearity was 0.995 from 20 kPa to 50 kPa. This section showed the highest sensitivity and linearity among the sensing range of a minimum of 5 kPa to a maximum of 50 kPa; approximately 50 kPa, but it was linear in the specific range from 10 kPa to 50 kPa. Existing piezoresistive type sensors were highly sensitive from several Pa to several kPa. However, it showed low sensitivity in the sensing range of several tens to hundreds of kPa. In addition, it was easy to receive damage from harsh pressing conditions applied to several tens of kPa. In contrast, the conducting nanomembrane-based pressure sensor revealed that Pearson’s R value was about 0.999, enabling linear sensing up to 50 kPa and becoming highly deformable (Figure 4e). This means that, unlike the existing piezoresistive-type sensor, linear measurement was possible even with the poked pressing of a small-diameter object. We have shown that the device can be operated repeatedly, which shows that this device can be used in applications such as HMI. Figure 4f shows the cyclic press-and-release test result of the cell. Pressing test conditions are the same as in Figure 4e, and even after repeating the operation for a total of 300 times, only approximately 1 ohm was shifted compared to the initial resistance. Figure S3 shows the same tendency of the resistance value rises slightly even in about 1000 cyclic tests. As a result, the prototype pressure sensor manufactured through the design rule has linearity and reliability among the high-pressure range. Based on the above, it was scaled up to a 16-channel array.

### 3.2. Pressure Sensing Demonstration

**Figure 5:**
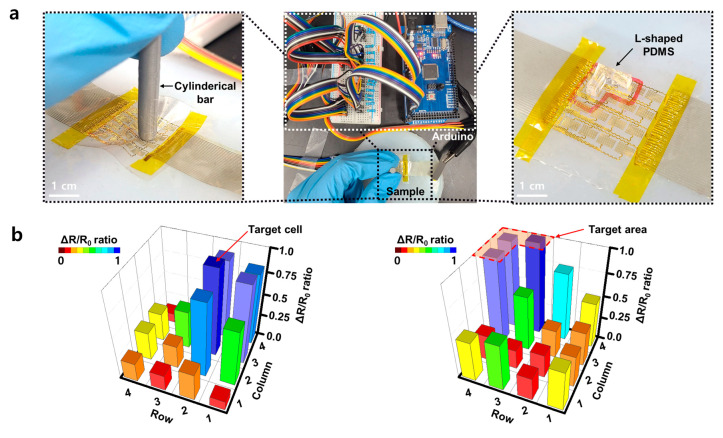
(**a**) A photograph of the array measurement system (middle) and a close-up photograph of pressure sensing at one cell (left) and a few cells (right) in the array (Scale bar: 1 cm). (**b**) Relative resistance change mapping graph of all cells when pressing one target cell (left) or target area (right) among 4 × 4 array cells.

Figure 5 shows the possibility of implementing the precise pressure sensing by using the sensor array. Figure 5a shows a 16-channel pressure sensor array measurement system. The reference resistor and the array sample were connected in series to ATmega 2560 (Arduino co., Boston, MA, USA) and the resistance was measured by the voltage dividing method. In the same way as the prototype sensor, the measurement was carried out by placing the sample on EcoFlex with a similar skin to Young’s modulus and pressing the sample with a cylindrical bar 3 mm in diameter (Figure 5a, left). Pressing a cell located in the second row and third column of the 4 × 4 array caused a deformation in adjacent cells.

Also, when several cells of the sensor were pressed with an L-shaped PDMS (Dow Chemical co., MIC, MI, USA) object for multi sensing, the deformation near the target area was detected again. However, as the distance from the targeted cell increased, the effect of deformation was rapidly reduced. Therefore, the nearby point pressed by an object or a human finger can be clearly measured (Figure 5a, right). Figure 5b shows that cells located near to the target cell had a ΔR/R_0_ ratio of about 0.87, but cells farther away show a significantly lower ΔR/R_0_ ratio below 0.5. In the array device, when the target cell or area was pressed, the end of the SEBS was lifted and the deformation of the cells at the corresponding position occurred, yet the pressed position and area can be clearly sensed from the ratio difference.

## 4. Conclusions

We fabricated a highly sensitive and soft, passive pressure sensor array, with linearity in wide range, by using simple microfabrication. The sensor can be attached to the skin as an electronic mechanoreceptor due to its stretchability. The fabrication method of the sensor is a simple process that is fully compatible with the semiconductor process. We fabricated an array of sensors by optimizing their sensitivity, which varied depending on the number of serpentines and the thickness of the substrate. This soft pressure sensor array was shown to be measurable from approximately 2.3 kPa to 50 kPa with linearity.

## Data Availability

The data presented in this study are available in this article.

## Supplementary Materials

The following are available online at https://www.mdpi.com/article/10.3390/mi12080933/s1, Figure S1: Relative resistance change for various numbers of sensing serpentine lengths, Figure S2: Relative resistance change for various thicknesses of SEBS substrates, Figure S3: Cyclic test of the targeted pressure sensor and close-up view of cyclic pressure sensing at middle range (inset).
