# Supplementary material for: A Soft Pressure Sensor Array Based on a Conducting Nanomembrane
Source: Micromachines (Basel). 2021 Aug 6;12(8):933. doi: 10.3390/mi12080933 (PMC8398079; doi:10.3390/mi12080933)
Supplement: Supplementary file 1 [file micromachines-12-00933-s001.zip › micromachines-1327319-supplementary.pdf]

Supplementary materials for

A Soft Pressure Sensor Array Based on a Conducting Nanomembrane

Daekwang Jung [1,†], Kyumin Kang [1,†], Hyunjin Jung [2,†], Duhwan Seong [1], Soojung An [1], Jiyong Yoon [1], Wooseok Kim [1], Mikyung Shin [3,4], Hyoung Won Baac [1], Sangmin Won [1], Changhwan Shin [1,*] and Donghee Son [1,4,5,*]

[1] Department of Electrical and Computer Engineering, Sungkyunkwan University, Suwon 16419, Korea; biglight0308@gmail.com (D.J.); zseqqq@gmail.com (K.K.); dodoworld1993@gmail.com (D.S.); soojung2134@gmail.com (S.A.); jiyong428@g.skku.edu (J.Y.); wooseokkim@skku.edu (W.K.); hwbaac@skku.edu (H.W.B.); sangminwon@skku.edu (S.W.)
[2] School of Mechanical Engineering, Sungkyunkwan University, Suwon 16419, Korea; hyunjin.jung33@gmail.com
[3] Department of Intelligent Precision Healthcare Convergence, Sungkyunkwan University (SKKU), Suwon 16419, Korea; mikyungshin@g.skku.edu
[4] Center for Neuroscience Imaging Research, Institute for Basic Science (IBS), Suwon 16419, Korea
[5] Department of Superintelligence Engineering, Sungkyunkwan University, Suwon 16419, Korea
* Correspondence: cshin@skku.edu (C.S.); daniel3600@g.skku.edu (D.S.)
† These authors contributed equally to this work.

Supplementary note 1:

At the beginning of the fabrication process, the wafers were immersed in acetone and isopropyl alcohol (IPA) and then surface-treated by bath sonication for 5 min. To ensure that the PI/metal/PI electrode, which was later manufactured, could be easily separated from the wafer substrate, the wafers were subjected to reactive ion etching with O_2 for 20 sccm at a pressure of 100 mTorr with 300 W of RF power for 5 min. After surface cleaning treatment, the PI precursor solution was spin coated at 7,000 rpm for 60 s and the PI-coated wafer was baked at 250°C for 1 h after curing it for 10 min and ramping it from 150°C. The Cr/Au metal layers were deposited using a thermal evaporator with thicknesses of 6 and 60 nm, respectively. After the spin-coated positive photoresist (PR) was soft-baked at 95°C for 90 s, the metal electrode patterns were left under the process of photolithography using a mask aligner with 200 mW of RF power for 10 s. Au and Cr etching were performed in order after developing for 1 min and 28 s, respectively. Then, the wafer was immersed in acetone to remove the undeveloped PR. The top PI layer was then spin coated, before the entire PI/metal/PI layer was patterned using RIE with O_2 at a 50 sccm flow rate, 100 mTorr pressure, and 450 W RF power for 10 min. An Al layer with a thickness of 100 nm was deposited as an etching mask to protect the PI layer below.

Supplementary note 2:

The sensitivity of the pressure sensor is defined as the ratio of the relative changes in electrical properties such as capacitance, resistance and inductance. The sensitivity of the piezoresistive pressure sensor is defined as:

$$S = \frac{\delta(\Delta R/R_0)}{\delta(p)}$$

where S is the sensitivity of the piezoresistive pressure sensor. The relative resistance change is expressed by $\Delta R/R_0$ where ΔR is the resistance change of piezoresistive pressure sensor and R_0 is the initial resistance of piezoresistive pressure sensor. The applied pressure is p.

Supplementary note 3:

Among existing papers, serpentine-designed polyimide (PI) within an Au thin-film layer has been studied as a 3D pressure sensor by Kwak, JW; Han, M.; Xie, Z.; Chung, HU; Lee, JY; Avila, R.; Yohay, J.; Chen, X.; Liang, C.; and Patel, M. et al. Wireless sensors for continuous, multimodal measurements at the skin were interfaced with lower limb prostheses. *Sci. Transl. Med.* **2020,** 12.). This paper suggested that serpentine-patterned PI encapsulated the Au nanomembrane, and that pressure sensing was possible up to several hundred kPa, respectively.

Polyimide (PI) patterned with serpentine design is a structure that encapsulates the gold nanomembrane. The actual device consists of a <3 μm thick PI structure on a 150 μm thick SEBS substrate, and both the sensing area and the interconnect are designed in a serpentine structure (Figure 3). In other words, the mechanical stiffness of the 150 μm thick SEBS substrate is greater than PI, and the serpentine-designed sensing area and interconnect stretch or return according to the deformation of the SEBS substrate. That is, when pressure is applied, the transferred PI device stretches along the SEBS deformation. At this time, the resistance value increases as both the sensing and the interconnect serpentine are stretched. Furthermore, resistance change is dominant as the 60 μm wide sensing serpentine is longer and narrower than the 150 μm wide interconnect. Due to this, pressure sensing based on serpentine structure is possible, based on the resistance change of the sensing area, while ignoring the resistance change of the deformed interconnect. In addition, the encamped PI provides stress dissipation to secure the ductility range (up to 0.8 %) of gold against high pressure, enabling repeated sensing [40].

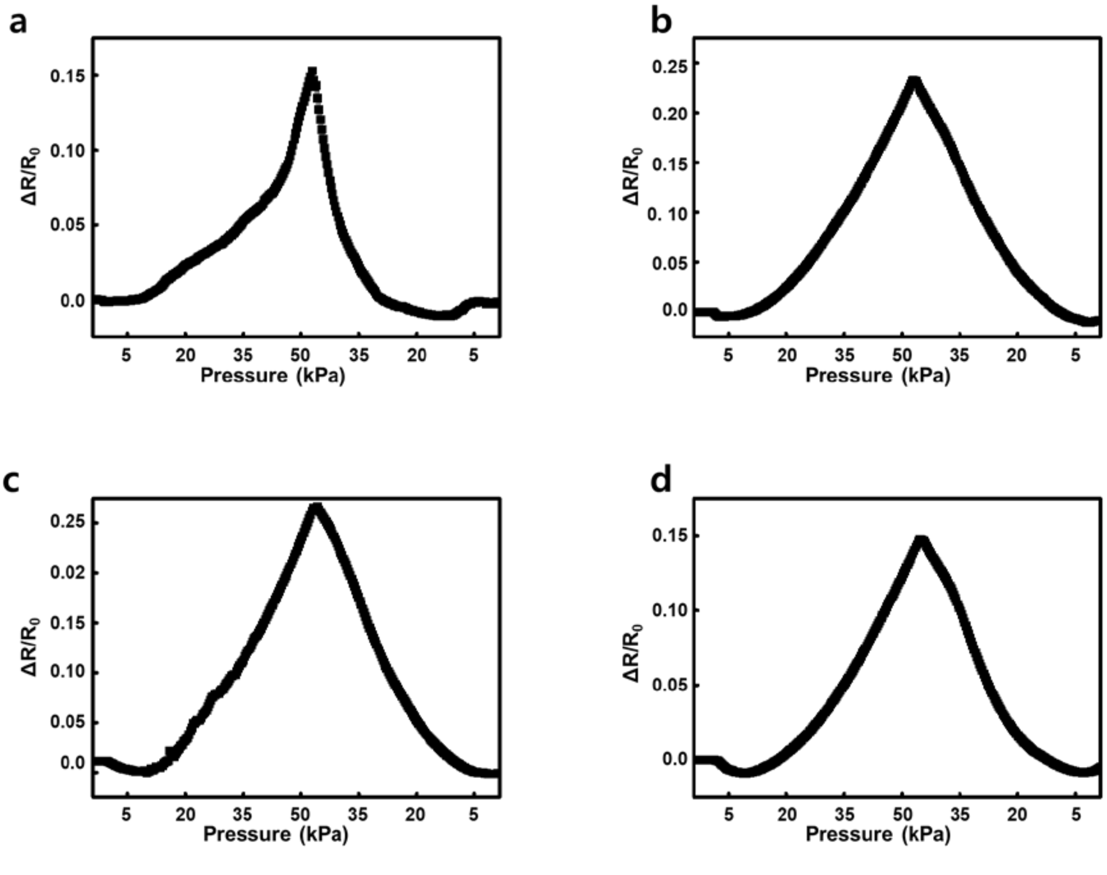

Figure S1. Relative resistance change for various numbers of sensing serpentine lengths. (a) ×2 patterns, (b) ×3 patterns, (c) ×4 patterns, and (d) ×5 patterns of sensing serpentines.

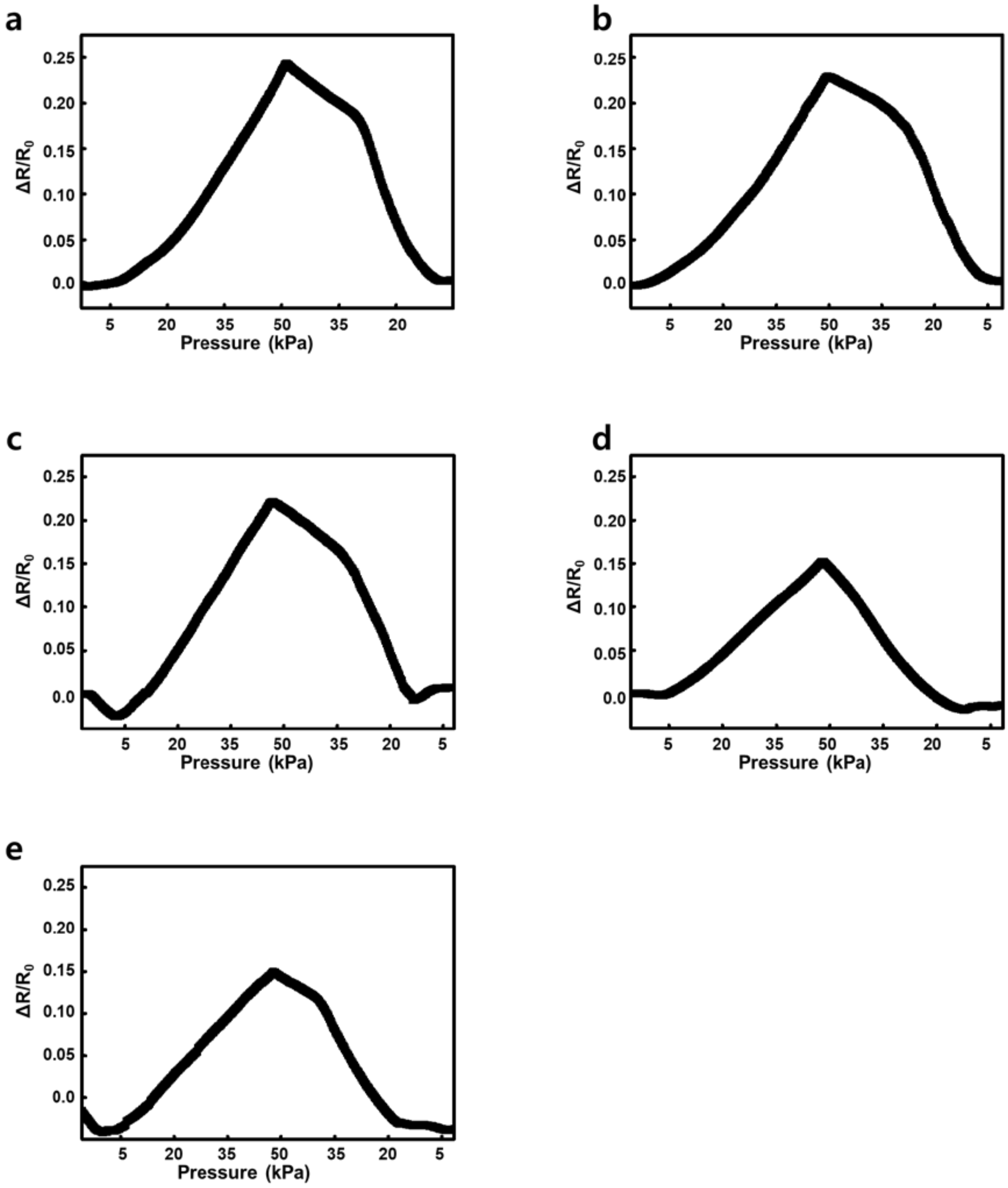

Figure S2. Relative resistance change for various thicknesses of SEBS substrates. (a) 50 μm, (b) 100 μm, (c) 150 μm, (d) 200 μm, and (e) 250 μm thickness.

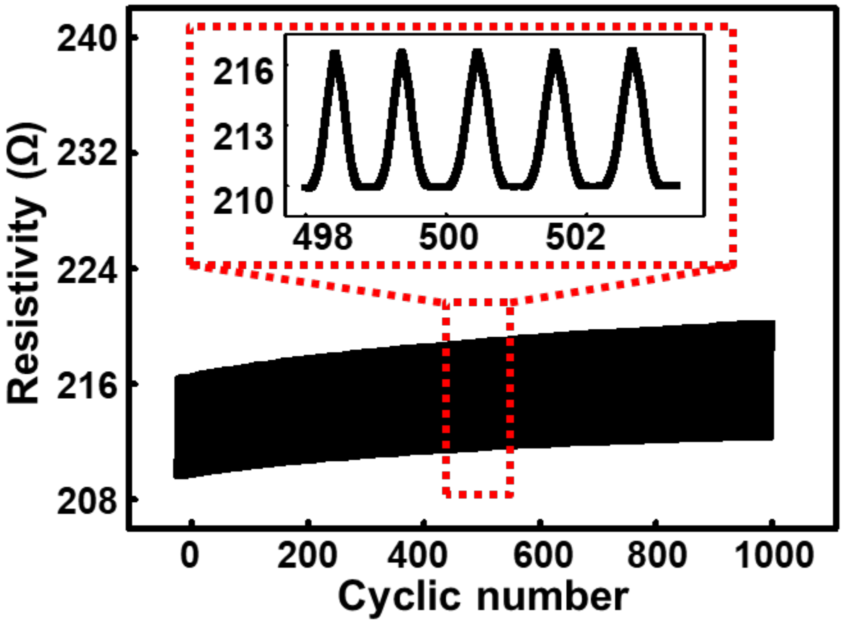

Figure S3. Cyclic test of the targeted pressure sensor and close-up view of cyclic pressure sensing at middle range (inset).
